# Methodology for assessment of public health emergency preparedness and response synergies between institutional authorities and communities

**DOI:** 10.1186/s12913-020-05298-z

**Published:** 2020-05-11

**Authors:** Daniel H. de Vries, John Kinsman, Judit Takacs, Svetla Tsolova, Massimo Ciotti

**Affiliations:** 1 Department of Anthropology, University of Amsterdam, Nieuwe Achtergracht 166, 1018 WV Amsterdam, the Netherlands; 2 European Centre for Disease Prevention and Control, Gustav III:s Boulevard 40, 169 73 Solna, Sweden; 3 Department of Epidemiology and Global Health, Umeå University, Umeå, Sweden; 4 Centre for Social Sciences, Hungarian Academy of Sciences, Budapest, Hungary

**Keywords:** Public health emergency preparedness, Community engagement, Synergies, Methodology, Protocol, European Union

## Abstract

**Background:**

This paper describes a participatory methodology that supports investigation of the synergistic collaboration between communities affected by infectious disease outbreak events and relevant official institutions. The core principle underlying the methodology is the recognition that synergistic relationships, characterised by mutual trust and respect, between affected communities and official institutions provide the most effective means of addressing outbreak situations.

**Methods:**

The methodological approach and lessons learned were derived from four qualitative case studies including (i) two tick-borne disease events (Crimean-Congo haemorrhagic fever in Spain, 2016, and tick-borne encephalitis in the Netherlands, 2016); and (ii) two outbreaks of acute gastroenteritis (norovirus in Iceland, 2017, and verocytotoxin-producing *Escherichia coli* [VTEC] in Ireland, 2018). An after-event qualitative case study approach was taken using mixed methods. The studies were conducted in collaboration with the respective national public health authorities in the affected countries by the European Centre for Disease Prevention and Control (ECDC). The analysis focused on the specific actions undertaken by the participating countries’ public health and other authorities in relation to community engagement, as well as the view from the perspective of affected communities.

**Results:**

Lessons highlight the critical importance of collaborating with ECDC National Focal Points during preparation and planning and with anthropological experts. Field work for each case study was conducted over one working week, which although limiting the number of individuals and institutions involved, still allowed for rich data collection due to the close collaboration with local authorities. The methodology enabled efficient extraction of synergies between authorities and communities. Implementing the methodology required a reflexivity among fieldworkers that ackowledges that different versions of reality can co-exist in the social domain. The method allowed for potential generalisability across studies. Issues of extra attention included insider-outsider perspectives, politically sensitivity of findings, and how to deal with ethical and language issues.

**Conclusions:**

The overall objective of the assessment is to identify synergies between institutional decision-making bodies and community actors and networks before, during and after an outbreak response to a given public health emergency. The methodology is generic and could be applied to a range of public health emergencies, zoonotic or otherwise.

## Background

Public health emergency preparedness (PHEP) refers to “*the capability of the public health and healthcare systems, communities, and individuals, to prevent, protect against, quickly respond to, and recover from health emergencies, particularly those whose scale, timing, or unpredictability threatens to overwhelm routine capabilities*” [1]. In this interaction between public health authorities and community members, it is clear that synergies benefit the eventual response and recovery after an outbreak occurs. The term ‘synergies’ here refers to the added value that derives from the process and outcome of two or more stakeholders or sets of stakeholders working together towards a common goal. Stakeholders could be either from the community, or they could be institutional. The benefits that are gained through working together are more than either could have achieved alone, and these benefits are, most likely, also mutually shared. ‘Community’ refers here to populations that may be directly affected by, or that may be at risk from the disease in question, or to networks of stakeholders who are linked to these affected populations and who may be able to assist in the process of solving or mitigating the problem [2]. Such actors may pre-exist in the civic or public realm in the form of stakeholder groups, each with their own interests, sub-cultures and specific expertise, or they may emerge during emergency events in the form of new coalitions. Overall, the community is seen as distinct from the government authorities who are formally and legally tasked with addressing the disease.

While there is a long history of interest in community participation in public health and development [3, 4], efforts to achieve synergies between authorities and communities within PHEP have until recently mostly been limited to socially disadvantaged groups [5–7] or focussed on barriers in governance [8, 9]. Observations have been made critiquing both the community engagement process and the outcomes of these efforts, and they have also highlighted the failed collaborations between anthropologists and outbreak response agencies [10]. Not until the 2014–2016 West African Ebola outbreak has the potential role of communities in PHEP become recognized more widely, specifically insofar as communities may be seen as collaborators whose capacities, insights, and skills need to be effectively incorporated into the response to public health emergencies [11–13]. Because successful community engagement processes are often hampered by institutional preferences for pre-packaged and top-down approaches, concerns have been raised about the sustainability of these efforts, while the need to understand broad assumptions about community capacity, motivations, interest to participate, and the reasons for lack of trust between agencies and communities has also been stressed [14–16].

In order for community-oriented PHEP efforts to be successful, it is necessary to understand how and the extent to which institutions in the health and relevant non-health sectors may collaborate with the community, and to identify good practices that have worked in one setting and that may therefore also be applied in others. This approach reflects the call by the 2015 Sendai Framework for Disaster Risk Reduction for a broader, more people-centred preventive approach through engagement with all relevant stakeholders [17]. Similarly, the need for the public sectors, civil society organizations, and scientific research institutions to work together more closely and to create opportunities for collaboration is also emphasized in EU Decision 1082/2013/EU (October 2013) on serious cross-border threats to health [18]. In this context, the question has emerged what the most effective way is to assess such synergies in order to provide information about good practices to interested stakeholders.

The aim of this paper is to detail and reflect upon an assessment methodology that can be used to identify potential or actual synergies that may have emerged between affected communities and relevant official institutions, prior to, during, and after infectious disease outbreak events. We found no previously published accounts of methodological approaches for conducting assessments of community-government synergies in PHEP. This paper contributes to the development of an approach that helps to identifying actual and potential synergies, thereby facilitating trust and effective collaboration between institutional authorities and affected communities before, during and after public health events. The approach described has been inspired on previous experience conducting after event assessments of disease outbreaks [19, 20] and experience with ethnographic research on community health resources [21, 22].

In order to describe and reflect upon the overall approach described, we collected information about the process from formal study reports, notes made by the researcher, and through discussions and reflections with those involved in implementation. The period of observation started in 2016 with the initial development of a literature review that was conducted to identify enablers and barriers to community and institutional synergies in emergency preparedness [23, 24]. After this, case studies were conducted in four EU/EEA countries in 2017 and- 2018: Spain, the Netherlands, Iceland and Ireland. The aim of these studies was to obtain good practices regarding PHEP synergies between institutional authorities and the communities studied. Two studies involved tick-borne disease events, both of which took place in 2016: Crimean-Congo haemorrhagic fever (CCHF) in Spain, and tick-borne encephalitis (TBE) in the Netherlands [25–27], and two outbreaks of acute gastroenteritis which took place in 2017–2018, verocytotoxin-producing *Escherichia coli* (VTEC) in Ireland, and norovirus in Iceland. All case studies dealt with relatively small events in terms of case numbers. These studies were conducted in collaboration with the national public health authorities in the four countries and with the European Centre for Disease Prevention and Control (ECDC). The methodological approach of these case studies was influenced by previous experience with after event assessment by ECDC on Middle East respiratory syndrome and polio [19, 20, 28].

## Methods

### Overall approach

The standardised study methodology described relates to an *after-event* assessment: it was developed to provide a basis for similar work in other settings. The overall objective of the assessment is to identify synergies between institutional decision-making bodies and community actors and networks before, during and after an outbreak response to a given public health emergency. The analytical framework chosen to organise the study involved the preparedness cycle [29]. Within this framework, the pre-incident phase involves preparation and planning; the incident phase involves management, monitoring, investigation, and intervention; and the post-incident phase involves recovery and identifying lessons learned. The assessment focuses on the specific actions undertaken by the participating countries’ public health and other authorities in relation to community engagement, as well as the view from the perspective of the community. The methodology is generic, process oriented, and could be applied to a majority of public health emergencies, zoonotic or otherwise. Overall, the approach can be characterized as a Grounded Theory approach [30], which sets out to discover or construct theory from data that is systematically obtained and analysed using comparative analysis in an iterative manner.

### Preparatory phase

#### Planning

Table 1 presents a Gantt chart for a hypothetical after-event assessment based on the methodology presented. Each case study would be expected to take approximately 8 months to complete.

**Table 1:** Gantt chart for a hypothetical after-event assessment

	Months							
	1	2	3	4	5	6	7	8
Project management	X	X	X	X	X	X	X	X
Fieldwork team meeting	X						X	
Customization of approach to specific case	X	X	X					
Literature review			X	X				
Fieldwork					X			
Reporting					X	X	X	X

#### Site selection and participants

A threat identification was conducted by ECDC experts on the basis of formal documents describing the events in the countries that had expressed interest to collaborate after informal contact and conversations. To enable good recall of the events and the lessons learned, it was decided that the outbreaks to be investigated should have taken place within the previous 3 years. Other criteria included the level to which the event had involved an inter-sectoral response, its community impact, biosafety level of the event, cross border issues, and the proposed study timeline. The eventually selected case studies together describe a wider variety of disease outbreak experiences according to the typology shown in Table 2. The typology is based on the extent to which the event was expected by involved stakeholders relative to the biosafety classification of the disease, the latter according to Directive 2000/54/EC of the European Parliament [31]. The typology suggests that the core principles identified are likely relevant to many other disease categories, including larger events, that fall within this typology.

**Table 2:** Typology of outbreak events studied (^a^ Biosafety level I not mentioned in this table includes no containment and unlikely to cause disease)

	Biosafety classification^a^		
	II (containment, moderate risk)	III (high containment, serious/potentially lethal)	IV (maximum containment, life-threatening disease)
**Expected**		VTEC (Ireland)	
**Surprising**	Norovirus (Iceland)	TBE (Netherlands)	CCHF (Spain)

After the countries and disease outbreaks were selected by ECDC experts, four or five teleconferences were organised with each participating country prior to data collection. This ensured that the respective needs of the participating countries represented by the National Focal Points for Preparedness and Response (NFPs), based within the national public health authorities, and the ECDC team were considered, and that the various logistical issues that needed to be worked through were all addressed. In coordination with the respective NFPs, a set of possible respondent categories was then identified. Depending on the nature of the disease outbreak and the country, these included stakeholders at multiple levels, including both national and county/regional/municipality level, as well as from both the health sector and relevant non-health sectors. Specific efforts were also made to include stakeholders with knowledge of any cross-border dimensions to the event. Possible respondent categories included were drawn from:

- *Professionals at national level*: Ministry of Health/Department of Public Health, State Epidemiology office, hospitals, epidemiological surveillance, risk communication, environmental health, occupational health, police and other emergency services, civil protection, education, agriculture, nature conservation organisations, patient organisations, and media;
- *Professionals at county/regional/municipality level*: Municipal/provincial authorities, local public health authorities, primary health care, education, food supply/control, agriculture, nature conservation organisations, environmental health, and media;
- *Affected communities*: The communities selected depended on the particular public health threat, but the focus would be specifically on communities at risk, and on vulnerable populations. Community leaders, representatives from interest groups, and civil society organisations were among those included.

The application of the method varied somewhat across all sites depending on health system characteristics and logistical needs. For example, in the first case study, Spain, the decentralized health system required more coordination between people at multiple levels, while language issues were a bit harder to overcome. In Iceland, on the other hand, the interconnectedness of the actors involved allowed for much easier shifting between levels. In the Netherlands more time was taken for the study because two diseases were included (Lyme Borreliosis and Tick-borne encephalitis) and the consultant team originated from the same country allowing more flexible scheduling.

#### Study design and sources of evidence

A qualitative case study approach [3] was taken, based on four data sources: (a) documents; (b) key informant interviews; (c) Focus Group Discussions (FGDs); and (d) participatory stakeholder mapping. Details of each of these data sources are given below.

- *Evidence source: Documents*

For each case study, a documentary review and analysis were conducted to provide background understanding as well as to facilitate the selection of groups and identify specific study questions. Documents included government, EU, WHO and other official materials on the respective events, provided by the participating countries, and supplemented, as available, by the peer-reviewed literature. The diseases were viewed through the lens of the analytically relevant preparedness cycle, and documentation from all three phases was specifically sought. The documentary review sought to identify, for all participating countries: (i) Policies concerning the prevention of the public health threat in question; (ii) Available standardized information on the capacity for public health emergency preparedness, response and recovery; (iii) Reports concerning challenges faced in preventing, diagnosing, and treating the public health threat; and (iv) Lessons learned from any simulation or training exercises on the public health threat, as well as from actual cases and events. Where necessary, documents in a language that the study team collectively did not know were put through translation software (e.g. Google Translate) in order to allow us to understand the main issues. For important issues that were found in the reports, interpretations of the documents were confirmed with respective NFPs.

b. *Evidence source: Interviews with experts*

Face-to-face Interviews were conducted during the five days of intensive, in-country fieldwork as a means of investigating actual experiences and individual perspectives of events. In many instances, these interviews included two or perhaps three peer assessors who worked closely together and whose experiences were similar or complementary. Interviewees were identified and recruited by the NFPs, based on the interviewee categories that had been agreed upon. In one case study—Iceland—cultural expectations regarding group work in a close knit social network motivated larger than usual interview groups. While this made the boundary between focus group and interview at times unclear, the dynamics that were captured reflected the actual working culture, and this was not seen as a disturbance to the quality of data collection. The questions were designed to be broadly relevant to all interviewee categories, but the focus of the questioning varied according to the position and experience of each individual interviewee.

c. *Evidence source: Focus Group Discussions*

Focus Group Discussions (FGDs) are valuable tools for investigating shared norms and understandings of an event. We used FGDs with somewhat homogenous groups of professionals (e.g. nurses) or community members (e.g. people working in nature areas), and/or in order to investigate community perspectives of an event. For all FGD, we tried not to mix people from different rank and seniority. The FGDs generally consisted of approximately 5–7 individuals each, although in some cases they were larger. Depending on logistics and availability, we aimed to conduct up to three FGDs over the course of each country visit. In some cases (e.g. Spain), translation was required.

d. *Evidence source: Stakeholder mapping*

Stakeholder mapping aimed to provide a retrospective understanding of the connections and relationships between the various categories of actors during the event, and it facilitated an assessment of engagement and interoperability between different stakeholders and sectors. In order to produce the stakeholder maps, interview and FGD participants were asked to draw out on a blank piece of paper the different stakeholder/interest groups that, from their point of view, they were involved with in preparing for and responding to the public health event under discussion in their country. This included both established or any newly emerging groups that became significant during the events studied.

#### Interview instrument

Using a systematic literature review that was conducted to identify enablers and barriers to community and institutional synergies in emergency preparedness [23, 24], a basis was laid for the interview guide. The literature review had involved organizing material into the context of a theoretical preparedness cycle, based on the pre-incident, incident, and post-incident phases [32], and the same framework was used to develop the interview instrument.

Two draft sets of questions were developed, one for professional experts, and one for community representatives. Both interview instruments started with a participatory mapping exercise. The draft interview and FGD guides were sent to the NFPs at least 2 weeks before each country visit in order to obtain their input and resolve any questions before field work began. If needed, the instruments were translated into the local language as appropriate for dissemination by the NFPs to participants before the interviews.

The example in Additional file 1 – produced for the case study on Crimean-Congo haemorrhagic fever in Spain – is for professionals from the health and non-health sectors. The questions are concerned with community-institutional synergies in the context of the specific tick-borne diseases under investigation. They could, however, easily be adapted to a range of other public health concerns.

Recruitment of study participants for the interviews, FGDs and stakeholder mapping exercise was undertaken by the NFPs and their teams. Based on the documentary review, a preliminary list of suggested groupings of potential study participants was shared with the NFP who further identified participants. Criteria for inclusion included diverse perspectives, different organisational levels, and relevant experience with the studied event. Typically, we received a full schedule of the interviews from the NFPs in the respective countries a week or so before field site visits, so we knew what would be happening, and when, on each day of field work. Each week-long country visit included around 10 individual interviews and three focus group discussions, giving us the voices of approximately 25–30 individuals for each country.

### Fieldwork phase

#### Fieldwork team

Five-day working visits were made to each participating country. Each country team included two people, including one senior social scientist with experience in qualitative data collection, and one junior social scientist who took extensive notes of what was said directly onto their laptop, made sure all details and materials were collected, and provided additional input in the interview process. Data were not audio recorded. First of all, this allowed for a more informal and trusting conversation, as audio recordings can raise the sense of formality of a discussion. In addition, by only taking notes we avoided the burden of transcription and were able to conduct fast analysis. Because it is important to be well informed in order to be taken seriously as an interviewer, the core research team prepared themselves by studying both the respective country health systems and public health aspects of the disease threat under study. During the first 2 days of each fieldwork period, ECDC experts joined the core team, which was important for ensuring proper introductions to the country authorities and for legitimising the study team’s presence. The core team was also joined by the NFP or a close colleague over the course of data collection, which created the opportunity for national level experts to engage in local level discussion, providing a valuable ongoing and iterative process of reflection.

#### Data collection approach and focus

A standardised field work guideline document – shown Additional file 2 - was prepared and shared with all fieldworkers. Preparation for each day of interviews involved close familiarization with the questions to be asked during the interviews and FGDs to be conducted that day. It was important for the interviewer to understand why all the different questions were being asked of each interviewee, in order to pursue interesting avenues should the discussion lead there. Interviewers also conducted some online research about the interviewees and the organization they worked in advance to help frame the questions.

Participants received the questions in advance of visits. For this reason, the interviews were conducted in a conversational manner, and not simply as if we were following a checklist of questions: every effort was made to ensure that interviewees felt at ease. Interviews/FGDs generally lasted for up to 60/90 min, respectively (although this was extended in the event of any translation requirements).

#### Post-interview debriefing

Post-interview debriefing sessions were held while travelling between interviews, or immediately at the end of the working day. Any interesting observations that either or both team members may have made were noted down; uncertainties that may have arisen in either of the team members’ minds about what happened in the interview were resolved; and key themes that may have emerged out of the interview were discussed. Because there were relatively few interviews and focus groups being held in each country, each interview was extremely valuable, and as a result the team ensured that any and all insights that had been gained were captured and recorded. Any quotes that were especially illustrative were highlighted in the notes, with an indication of who made them.

#### Hot debrief

A ‘hot debrief’ was held on the last day of fieldwork, including the team in-country (i.e. the NFP with their staff and the respective research team) and the relevant ECDC experts in Stockholm who joined by phone. These discussions constituted the first formal ‘think-through’ of the whole week’s work, including some of the main themes and issues that the study team wanted to highlight in the respective country report, and as such were valuable moment for reflection.

#### Ethics and data security

Written informed consent was obtained from all informants during site visits. During this process the objectives of the case study was explained to the interviewees, and they were assured of their right to withdraw from the interview/FGD at any time. Unless informants explicitly confirmed in writing that they were willing to go on record, their comments remained anonymous within any reports and/or subsequent publications resulting from the studies. All interview and field note materials were kept securely, and only the study team (which includes any ECDC staff who are involved) had access to it. We complied with Regulation (EC) No 45/2001 on the storage of personal data and on ensuring citizens’ privacy. ECDC was the data controller of this processing operation, even though the data were collected and stored by the implementing research partner. All interview and documentary material were handed over to ECDC at the conclusion of each case study.

### Post-fieldwork phase

#### Analysis of observations from the study/assessment

For each country case study, the qualitative data collected from the participating countries were subjected to thematic analysis, using qualitative data software (e.g. OpenCode or NVivo). The findings were again placed in the context of the theoretical preparedness cycle, based on anticipation, response, and recovery phases, as identified above. To the extent that we were able, given the limited sample size for our interviews and FGDs, we also distinguished between the national and more local levels in the analysis. Where appropriate, quotes were included in the reports as illustrations of particular points, but these were anonymised in order to ensure confidentiality for the interviewees.

#### Stakeholder mapping

**Fig. 1:**
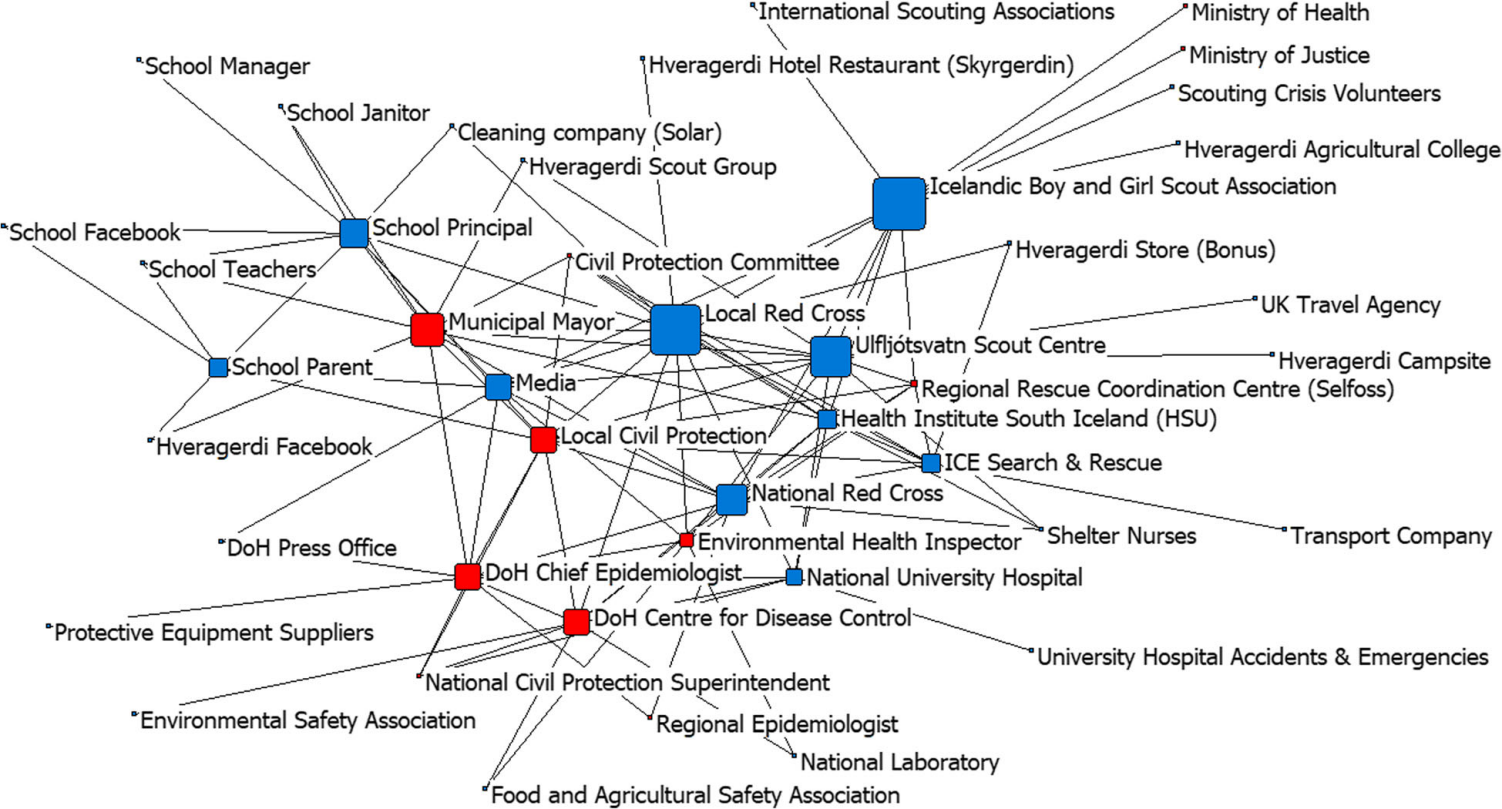
Social network of stakeholders mapped in the norovirus outbreak, Iceland^.^ Size of the nodes indicates degree of brokerage. Colour indicates either a government authority (red) or community-based actor (blue)

Data from individual respondents’ stakeholder maps were compiled into UCINET software, with symmetry forced into the matrix. The resulting social network was complemented by an attribute file that indicated whether the various stakeholders were government authorities or community-based, as well as their medical, educational, environmental, or animal health identities. After this, a measure of centrality, or “betweenness”, was used to quantify the number of times a node acts as a bridge along the shortest path between two other nodes [33]. This measure is intended to illustrate the control of the node on the communication between other nodes in the social network. These nodes are seen as “brokers” of information because they connect otherwise disconnected nodes. As a result, they are also the ones whose removal from the network will most disrupt communication between other nodes because they lie on the largest number of paths taken by messages. Figure 1 shows an example of a resulting stakeholder map.

#### Review process

The country case study report was delivered in stages. First, the entire ECDC team involved reviewed the product for a first round of revisions. After this, the revised document was sent to the country NFP for their input, with as wide a dissemination as the NFP deemed desirable. After their input, the revised version was again submitted to ECDC for formal clearance, and finally approved by the ECDC Chief Scientist. This process ensured that input was obtained from a range of experts as well as country-level stakeholders, and that the final product represented a fair and accurate summary of what had been found. All reports included details of the main findings on good practices and challenges with regard to synergies between institutional and community-level actors, including with regard to cross-border and inter-sectoral collaborations. Country reports also included a focus on national level strategic planning issues and, as appropriate, of operational and implementation factors at local level.

Two combined disease-specific reports were also produced, based on a synthesis of the individual country-level reports: one for the tick-borne diseases, and one for the gastro-intestinal diseases. These included a compendium of actions and good practices taken by institutional and community stakeholders in the participating countries.

## Results

The described methodological approach described has been used for after-event review of four infectious disease outbreaks across the EU/EEA. We will here consider some of the lessons learned and implications of this approach. The literature review and case studies served as the basis for the development of the approach described. However, additional reflections on the methodology and process were made during an expert consultation held in at the ECDC offices in Stockholm on March 27/28, 2019. Participants at this meeting included 15 international community engagement actors, technical experts, and ECDC stakeholders, including four National Focal Points for Preparedness and Response^1^ (not from the countries involved in this study). ECDC staff members also participated.

### Extracting synergies

The extraction of good practices for promoting collaboration and synergies was conducted during several phases in line with the grounded theory approach. The process of extracting potential synergies already started before fieldwork through the literature review [34] and by analysis of the documents provided by the countries. These were seen as first indications and potential avenues for further discussion, but an open mind was intentionally kept before fieldwork. During the fieldwork phase the core team was joined by the NFP or a close colleague representing the authorities over the course of data collection. The chance for this person to engage in a four-day dialogue with community partners was often experienced as enriching for all parties involved and created in-situ opportunities for synergies to develop or the identification of barriers that had previously not been recognized. The opportunity to ethnographically reflect upon and document this experience further helped to identify possible or actual synergies. During the “hot debrief” the last day of fieldwork, the dialogue between authorities involved, the fieldworkers, and ECDC stakeholders typically allowed for a limited prioritisation of the most relevant findings. In the post-fieldwork phase, many of the good practices obtained were directly derived from documented comments made by the respondents. These comments were typically contrasted with results from the stakeholder mapping exercise to further contextualize social positioning of a stakeholder among all the established or emerging actors. In addition, a special team meeting was organized near the end of the project to prioritize, contrast and compare all the identified synergies from the different case studies in an overall, cross-case study set of good practices. In the final phase, the extensive review process helped to flesh out unclarities and point out missed synergies.

### Reflexivity

One of the unavoidable results of conducting qualitative case studies among a diversity of respondents that include both government and community perspectives is that qualitatively different perspectives on the same outbreak will be provided. While it may be tempting to try to find out the “right” perspectives, often different versions of reality can co-exist in the social domain. With regard to the assessment of community engagement, it is crucial that all the versions of events promoted by different community actors are taken seriously, as discourses and ideas determine social action as much as – if not more than – hard, scientific facts [35]. This “constructivist” perspective suggests that the goal of the assessment should not be to delineate the “correct” from the “false”, but rather to compare and contrast different versions – also referred to as “frames” – of what happened, identifying agreements and disagreements, and how these may have led to particular actions during an outbreak. In the context of emergency response this is not always without challenges, as medical professional culture is generally inclined to favour a positivist scientific approach in which factual, biomedical knowledge is perceived to be the only “correct” way of understanding disease outbreaks events. For researchers in the field, this situation therefore demands proper training in social science techniques. Central is the ability to be reflexive about the fieldworkers’ own values and position, in order to distinguish a researcher’s own biases and assumptions from those of the voices obtained in the field [36, 37]. Why do we see a phenomenon one way or another? What biases and assumptions determine how we look at an event? Recognition of the need for such questions is inherently an acknowledgement that the orientations of researchers will be shaped by their own socio-historical background.

An example of a reflexive attitude is to always maintain an open attitude about what “community” actually is when in the field. As there is no one way of seeing and defining “community”, the entire idea of community engagement during an outbreak also may shift according to context or situation. It is this type of sensitivity which is necessary to conduct quality data collection and analysis. It demands reflexivity not only towards oneself, but in the entire methodology of the assessment. Moreover, such reflexivity helps to find sensitivity when perspectives on the same event are in disagreement and group tensions become visible. For example, it is not unlikely that some people may be upset by the findings – e.g. the authorities will say that the community doesn’t appreciate their work, or the community will say that the authorities never listen. This creates an important but challenging divergence of views, and it is here that a social scientist will move in to try to disentangle what has happened through using reflexivity and sensitivity in how they present the findings.

### Assessment

The relatively short time available for data collection in the field does limit the number of individuals and institutions involved in the study, and possibly therefore also the range of issues that can be covered. For this reason, the significance of working with the ECDC NFPs in advance of and during the country visits is crucial to develop an effective and efficient program. The NFP identifies the most relevant interviewee and FGD participants, as well as the key documents, which consequently maximises both the range and quality of the data collected within the time constraints. The disadvantage of this approach is of course the possibility that depending on the social network of the particular NFP, some respondents may be left out or not. A possible remedy here is to conduct a more formal stakeholder analysis not dependent on the experience of the focal point to support the selection of respondents previous to fieldwork. However, because we asked each participant to briefly draw an informal map with everyone they had interacted with, we relatively quickly identified groups that had not been mentioned or listed, and were able to make last-minute additional appointments or inquiries if necessary. It is well known that during disease outbreaks certain groups of actors may emerge as relevant that were before not recognized. Our participatory mapping was able to quickly identify these groups.

Overall, we believe that for the studies reported on here, the core themes under investigation were generally very well covered during the five-day country visits, including from a range of different perspectives, even though some key respondents were missed as a result of the study limitations. Further, the complementary semi-structured qualitative interview and focus group methodologies that we adopted were sufficiently flexible to allow participants to discuss issues as they wanted to, and to raise topics that they felt were important, thereby opening up the possibility for us to document points that we had not known or thought about in advance. We are therefore confident that while we may not have achieved saturation on every relevant theme, the major issues were all addressed in sufficient detail to produce conclusions that were ultimately seen as relevant by the stakeholders who reviewed the findings.

### Generalisability of findings

The choice of case studies (i.e. both the disease and the participating countries) will naturally define the findings, and hence may also affect their potential generalisability to other settings. We recognise that the findings from each case study will necessarily be specific to the contexts in which we worked. We cannot expect that the findings from one country will be entirely applicable in another, where the health system and wider culture may be quite different. Overall, since the primary stakeholder for each case study was the country in question, this issue was not experienced as a problem. However, mapping the characteristics of the case studies out on a typology (see Table 2) helped overcome this limitation to some extent, as the exercise showed how the case study addressed characteristics that are generalizable to other disease categories and contexts. Previous experience from the multi-country ECDC case study work indicates that some findings may be sufficiently generic as to be potentially applicable in several countries. Further, as we conducted more case studies, overlapping themes started to emerge, leading to a fairly robust set of findings that built on this expanding dataset and that we believe is applicable for the promotion of community engagement in many if not most of the EU/EEA, candidate and/or European Neighbourhood countries.

### Insider-outsider perspectives

As outsiders, there were inevitably political and/or cultural issues in each country that we visited that we, as the research team, may not have fully understood. This likely affected our interpretation of the findings. Again, the close collaboration with the NFPs on the draft country reports we produced for the various case studies ensured that our findings were consistent with local norms and also that, at least according to the NFPs who reviewed our draft reports, we have not made any fundamental errors in interpretation. We believe that this combination of an outsider perspective being supported by insider knowledge can produce very strong findings. In addition, we generally used an anthropological approach in our field research, which builds upon professional experience and expertise with cultural diversity and openness to differences in interpretation of the same event. Furthermore, the same event can be interpreted differently by different insider stakeholders, which can give an outsider a unique perspective from which to coherently bring together occasionally contradictory observations.

### Burden on countries and use of results

Each country that participated in the assessment had a sense of co-ownership of the process motivated through the participatory methodology. Organising the assessment did take some extra time and the availability of a representative from the authorities was typically a concern. However, the rewards of participation appeared to be highly appreciated. Representatives of all the participating authorities voluntary joined a follow-up meeting meant to develop a guidance based on the findings, indicating their appreciation of the process.

### Politically sensitive findings

During the country visits, we learned of issues on some occasions that could be important but which were politically sensitive. Reporting these may be challenging, but we always sought to find a way of expressing such findings in a manner that says what needed to be said but that still maintained confidentiality or that otherwise protected the political sensibilities in question. This has been accomplished through discussions with the ECDC project officer and with the NFP of the country in question. We recognised that the Member States are primary stakeholder in these case studies, and we have always made every effort to ensure that we maintain their confidence throughout the whole process.

### Ethical issues

Ethical challenges may be encountered in this sort of after-action assessment, most likely during field work. Some respondents were concerned, for example, about their comments being passed on to superiors, and sensitive legal matters were also raised during some of our discussions. For this reason, it is very important to include experienced field workers as core team members who are all well acquainted with the Declaration of Helsinki as well as the associated bioethical principles that must always be adhered to during research (beneficence, non-maleficence, justice, and autonomy^2^). It is also important that the NFP and/or the ECDC project officers are available for discussion should there be any question about how to ensure that proper study protocols were followed.

### Language

While our core team has language competency in different languages, some communication challenges were experienced during the country visits. Some of the country documentations were also be in a language that we did not understand. This challenge proved to be surmountable. For understanding documents in non-English languages, we used Google Translate (where digital copies are available) – and while this does not give a perfect translation, it does nonetheless provide a good overview of the contents which we could subsequently clarify as necessary during discussions with the NFPs (all of whom speak excellent English). For translation during interviews and Focus Group Discussion, we have used different solutions at different times, including hiring a professional translator, or having the NFP or one of their co-workers act as an intermediary. Some nuances are inevitably lost when the process is mediated like this, but the major issues do come through and we have been able to document these.

## Discussion

We compared these findings of our approach to the well-known system of quality criteria for qualitative research developed by Guba, Lincoln and colleagues [38] which review general trustworthiness.

### Credibility

Credibility refers to the extent to which participants or members of the community being researched feel that the findings represented their experience. We believe that the methodology provided ample opportunity for input and inclusion of the various experiences of participants. These included a participatory design of the study whereby the NFPs (or representatives) were included in the fieldwork, a “hot debrief” at the end of the fieldwork period, and the opportunity for participants to provide feedback during the final review of the reports. In addition, a process of triangulation occurred by comparing and contrasting the documentary, interview, focus group, observational and social network mapping. We also included an extensive peer review trajectory. Still, and as noted above, the exercise also clearly showed us that there is not one “right” perspectives to be captured and that different versions of what happened in the outbreak event co-existed in the social domain. The comparison and contrasting of different frames of what happened could potentially lead to some participant feeling that their view is underrepresented.

### Transferability

Transferability refers to the extent to which the findings are applicable in other contexts. Because the methodology was largely qualitative, this is a more difficult aim to achieve (see also generalizability of findings above). Because we took notes instead of full transcripts and because the style of reporting within ECDC follows a biomedical instead of a social science tradition, the individual country case study reports did not provide the “richness” of descriptions common to ethnographic study and overall excluded the researcher’s reflexive interpretations. This made transferability of the findings each of the case studies harder to evaluate from the reports alone. However, richer descriptions can also be obtained with the methodology; the limiting choice of style in this case was institutional. For our own studies, we remedied this situation with two separate team meetings where the teams across case studies came together for 2 days to discuss and evaluate common points of good practice. In this process, discussions of interpretations were central, and we noted that robust, overlapping themes emerged, nevertheless. In addition, the mapping the characteristics of the case studies out on a typology (see Table 2) also helped overcome this limitation, as the exercise showed how the case study addressed characteristics that are generalizable to other disease categories and contexts.

### Dependability

Dependability refers to the extent to which similar findings would be produced if someone else undertook the research. Despite the disadvantage of qualitative research overall compared to quantitative surveys, we had confidence in the dependability of the results. The team approach allowed for an open dialogue where one researcher could not dominate the discussion. Instead, the results often came together through the voices of many, each contributing their part, and the triangulation of observations across researchers within the team afterward further increased the dependability of findings. Of course, the timing of the after event review does influence the dependability. Overall, we ensured that the outbreaks to be investigated should have taken place within the previous 3 years in order to avoid major memory recall problems. Still, political situations happening at the time of fieldwork likely influenced the outcomes to some extent; this is an unavoidable result of a non-longitudinal qualitative approach. To avoid this, a second fieldwork period could be considered.

### Confirmability

Finally, confirmability refers to the extent to which the findings a product of participants’ responses and not the researcher’s biases, motivations, interests, or perspectives. As already noted, the style of reporting did not allow for traditional richness of social science presentation, which also allows for evaluation of confirmability. We also did not conduct an auditing trail. However, we do believe the findings reflect authenticity and a fair range of differing viewpoints on the topic because the researchers took extra care to be sensitive to their own positionality and included a strongly reflexive approach common to anthropological inquiry. We also believe the findings had a transformative potential, because the dialogical approach during interviewing complemented by the inclusion of a representative from the authorities at each interview (who was there to listen and learn), helped to motivate community consensus towards the usefulness and meaningfulness of the findings for future action and further steps. The authenticity of the findings was only questioned by one stakeholder in one case study, and this situation seemed to revolve around unresolved coordination tensions with a longer history.

## Conclusion

The methodology presented in this paper relates to an *after-event* assessment using a largely qualitative research design. The overall objective of the assessment is to identify synergies between institutional decision-making bodies and community actors and networks before, during and after an outbreak response to a given public health emergency. ‘Synergies’ here refer to the added value that derives from the process and outcome of two or more stakeholders or sets of stakeholders working together towards a common goal. The analysis focuses on the specific actions undertaken by the participating countries’ public health and other authorities in relation to community engagement, as well as the view from the perspective of the community. The methodology is generic and could be applied to a majority of public health emergencies, zoonotic or otherwise. It highlights the importance of dialogue during preparation and planning, and interviewer reflexivity during fieldwork. The assessment can be done in a relatively short time, and while the design limits the number of individuals and institutions involved, the insider-outsider collaboration with local focal points allows for a rich fieldwork experience in which many of the relevant angles are still addressed. Comparing the outcomes with quality indicators for qualitative research, the outcomes generally show good trustworthiness. Despite the fact that results cannot easily be generalized across contexts, the emergence of key principles in community engagement that are relevant across contexts is nonetheless likely.

## Supplementary information

**Additional file 1.** Example of interview instrument.

**Additional file 2.** Guidelines for tick-borne disease case study: Spain and the Netherlands, October–November 2017.

## Data Availability

The interviews used and analysed for the current study were conducted with a range of government and provincial/regional level officials from the four participating countries, and as such the datasets cannot be made publicly available. However, ECDC may be consulted for further inquiry on availability.

## Abbreviations

CCHF: Crimean-Congo haemorrhagic fever
ECDC: European Centre for Disease Prevention and Control
FGD: Focus Group Discussion
NFP: National Focal Point
PHEP: Public health emergency preparedness
TBE: Tick-borne encephalitis
VTEC: Verocytotoxin-producing *Escherichia coli*

## Footnotes

1 The main mode for ECDC to interact on scientific and technical work with the Member States is within networks and working groups with members being nominated by their respective Competent Bodies. National Focal Points for Preparedness and Response are individuals identified by a country’s National Coordinator in the Competent Bodies or from other institutions of the Member States as delegated representatives for disease groups and public health functions, such as Preparedness and Response. (See: https://www.ecdc.europa.eu/sites/portal/files/media/en/aboutus/governance/competent-bodies/Documents/coordinating-competent-bodies-structures-terms-of-reference-and-interactions-w-Annexes.pdf)

2 https://www.wma.net/policies-post/wma-declaration-of-helsinki-ethical-principles-for-medical-research-involving-human-subjects/
