# Supplementary material for: Methodology for assessment of public health emergency preparedness and response synergies between institutional authorities and communities
Source: BMC Health Serv Res. 2020 May 11;20:411. doi: 10.1186/s12913-020-05298-z (PMC7212582; doi:10.1186/s12913-020-05298-z)
Supplement: Supplementary file 1 — Additional file 1. Example of interview instrument. [file 12913_2020_5298_MOESM1_ESM.docx]

**Additional file 1: Example of interview instrument**

Interview Questions for institutional representatives

**Part 1: Mapping the different stakeholder/interest groups**

1. Please tell us how you and the institution you work for have been or are involved with Crimean–Congo haemorrhagic fever (CCHF).

2. Could you map out on a piece of paper (a) the different stakeholder/interest groups or groups that have previously been involved with preparing for tick-borne diseases, and (b) those that were involved in responding to the 2016 CCHF cases. Which of these groups would you define as coming from the community, and which would you define as “Administration”? Do you think there are any stakeholder/interest groups – institutional or from the community – who are missing from this map, but who should be included in order to ensure better preparedness and response in future?

**Part 2: Issues arising during each of the three phases of the public health event**

Anticipation phase (prior to the event)

3. Has your institution produced any protocols, guidelines, or information leaflets for the population regarding the prevention of tick-borne diseases? [Obtain copies if possible]

4. To what extent were there any sort of public health preparedness activity or simulation exercises, consultations, or training activities involving both the community and the Administration prior to this case? Please describe these. Do you consider these activities to have been (a) necessary, and, if so, (b) sufficient? If not, what could have been done in addition?

5. [FOR NATIONAL LEVEL RESPONDENTS] In general, do you think that the community trusted the public health & scientific administration prior to the event? [FOR AUTONOMOUS COMMUNITY LEVEL RESPONDENTS] In general, do you think that the community trusted the public health & scientific administration in Castilla y Leon and Madrid AC prior to the event? [FOR ALL RESPONDENTS] Had there been any specific events (such as other disease outbreaks) that promoted or undermined trust? Details.

Response phase (during the event)

6. [FOR NATIONAL LEVEL RESPONDENTS] Were there sufficient numbers of dedicated professional staff, able to respond to the case? [FOR AUTONOMOUS COMMUNITY LEVEL RESPONDENTS] Were there sufficient numbers of dedicated professional staff in Castilla y Leon and Madrid AC, able to respond to the case? [FOR ALL RESPONDENTS] Were there any problems, for example with funding, that may have limited the response?

7. Was there any official guidance for the administration on how to engage with the community in this case(s)? What form did this guidance take?

8. Were the key actors in the community clearly identified and available when the case(s) first appeared? To what extent was there clarity about who was expected to do what?

9. What were people’s sources of information about the event (i.e. press and social media etc.)? How informative, coherent and consistent were these sources of information? Were there any issues that you think people felt they needed to know more about?

10. How was the communication and coordination between the community and the administration during the response to this event? [i.e. shared/transparent/top-down?]. Were there any aspects that could have been improved?

11. To what extent do you think different groups who could have been at risk within the community (e.g. hunters, farmers, mountaineers and hikers) cooperated with each other during the response to this event? Examples?

12. Do you think there were any groups in the community who, for any reason, were excluded from the response? Details.

13. Were there any hard-to-reach or vulnerable groups [PROBE: for example, undocumented migrants working on farms]? What efforts, if any, were made to reach out to them with information about prevention and, if necessary, treatment-seeking behaviour? Who led these efforts, and what lessons could be learned from this?

Recovery phase (after the event/outbreak)

14. Was there any sort of post-case review of the event, specifically with reference to the ways in which the community and the administration communicated and collaborated together? If so, what form did the review take, who was involved, and what was the outcome?

15. How much awareness do you think there currently is in the community about this event? Do you think that lessons have been learned by the community regarding prevention and response practices for future events of this nature?

**Part 3: Overview**

16. Overall, how would you rate (i) the community response and (ii) the official response to the event? Were you satisfied, or do you think some aspects could have been improved?

17. [ONLY FOR AUTONOMOUS COMMUNITY LEVEL RESPONDENTS] In general, how do you feel the community and the administration collaborated during this event? What would you say was the most successful aspect of any collaboration? What were the main challenges faced in the collaboration process, and what efforts, if any, were made to overcome these?

18. What do you think are the main lessons learned from this event, in terms of community- institutional collaboration and preparing for future public health emergencies or events?

19. Is there anything else you would like to add?
