# Supplementary material for: Methodology for assessment of public health emergency preparedness and response synergies between institutional authorities and communities
Source: BMC Health Serv Res. 2020 May 11;20:411. doi: 10.1186/s12913-020-05298-z (PMC7212582; doi:10.1186/s12913-020-05298-z)
Supplement: Supplementary file 2 — Additional file 2. Guidelines for tick-borne disease case study: Spain and the Netherlands, October–November 2017. [file 12913_2020_5298_MOESM2_ESM.docx]

**Additional file 2:** *- Guidelines for tick-borne disease case study: Spain and the Netherlands, October-* November 2017

These guidelines are for the team members, and they are intended to help standardize the approach to field work for the case studies in Spain and the Netherlands.

1. **Country teams**

Each country team includes two people, a country lead and note taker/fieldworker. Each interview will be led by one team member with note taker/fieldworkers taking extensive notes of what is said directly onto their laptops. Note that we will be accompanied to the interviews on the first two days of the country visit by ECDC staff; and we can also expect to have the NFP or their representative/s join us for many or most of the interview and focus group sessions.

#### Preparation before the field visit

It is important to be well informed in order to be taken seriously as an interviewer, both about the country health systems where we are working, and about tick-borne diseases generally. A comprehensive set of documents are placed on the shared project site, to which all field workers have access. Team members should carefully read and internalize the materials that will be posted on this page. The documentary review for the two participating countries will also assist in the preparation process.

#### Preparing for and conducting interviews and FGDs

*The interviewer*:

- It is important that we understand why all the different questions are being asked of each interviewee, so that we can pursue interesting avenues should the discussion lead there. Preparation for each day of interviews will therefore involve close familiarization with the questions to be asked during the interviews and FGDs to be conducted that day. We expect to receive a full schedule of the interviews from our colleagues in the respective countries before our visits, so we will know what is going to be happening, and when, on each day of field work.
- Some online research about the interviewees and the organization they work for could also be done in advance, as this may help inform the questions as well as our understanding of the answers. However, it is important to restrict this research to professional issues, and to avoid any mention during the interviews of personal issues about the interviewees that may inadvertently emerge through the searches.
- The interviewees will have received the questions in advance of our visits, so they will be familiar with the issues of interest to us. We should try, therefore, to conduct the interview in a conversational manner, and not simply as if we are following a checklist of questions: interviewees should be made to feel at ease. Ideally, we should not allow the interviews/FGDs to go on for more than 60/90 minutes respectively (though this may be extended in the event of any translation requirements). It is important that we are not seen to be imposing on people’s valuable time.

*The note-taker*

- Good note-taking is of paramount importance for this project, as the words that are written down during the interview, and then worked up later on the same day (see Point 6 below), will constitute much of the data that will be used for the report. The note-taker’s capacity to capture both content and nuance is therefore a core requirement for a good study outcome. The note-taker should be prepared for up to three to four hours of concentrated note-taking per day during the field visit.
- The note-taker should familiarise him/herself with the questions before each day, as well as the organizations to be visited – just as the interviewer will be doing.
- Although the lead team member will take the lead during the interview, the note taker is also welcome to ask questions as he or she sees fit.

#### Travel between interviews

Since we want to minimize inconvenience to our interviewees, most of the interviews will be conducted in their offices at the institutions where they work, and the focus groups will be held at a recognised community facility that is known to the focus group members. Therefore, we will spend a portion of each day travelling between interviews and focus groups. We do not know exactly how this will be arranged at this stage, but we should not expect that transport will be provided by our hosts. If transport is not provided, we will take public transport (i.e. bus or metro) where feasible, in order to minimize costs. Taxis should only be used if there is realistically no other option to get to an interview in time. Be sure to keep receipts in order to get reimbursed.

#### Post-interview debriefing

It is important that the team discusses each interview *as soon as possible after it has taken place*. Memories fade fast, even of really interesting points, and especially when there is a lot happening in a day – as will be during the field visits. These debriefing sessions should ideally take place while travelling between interviews, or immediately at the end of the working day. Any interesting observations that either or both team members may have made should be noted down; try to resolve any uncertainties that may have arisen in either of the team members’ minds about what happened in the interview; and agree on a couple of main themes that emerged out of the interview. This latter will be helpful in the analysis. With relatively few interviews and focus groups being held in each country, each interview is extremely valuable, and we must be sure to capture and record any insights that we have had before they are lost.

#### Interview notes and data security

*It is essential that all interview notes – both by interviewer and note-taker – are typed up and emailed to the other country team member and (for the Netherlands) to the PI at the end of each day.* This is both to ensure that the notes are worked up while the interviews are still fresh in interviewer’s/note- taker’s minds, thereby maximizing data quality; but also to ensure that the material is saved elsewhere in the event of a computer crash, loss, or theft. Interview notes should be as detailed as reasonably possible given the time available, and they should be checked through before being sent off so that there are no issues which we won’t be able to resolve later.

- The note-taker should send the cleaned interview notes plus any comments or insights that they may have had about the interview to the interviewer and (for the Netherlands) to the project PI.
- The interviewer should also make his own notes of the interview, including all observations and thoughts, and send these to the note-taker and (for the Netherlands) to the project PI.

It doesn’t matter if both interviewer and note-taker make similar comments – every impression is valuable.

*Any quotes that are especially illustrative should be highlighted, with an indication of who made them*. The name and other details will not be included in any publication, but we need to know who said what when writing the report, for the sake of context.

#### Final debriefing

Each field work team will have a debriefing session on the Friday afternoon of the country visit, with the national counterparts and ECDC (by skype or some other secure means). John K will join the Netherlands debrief by skype, and Daniel de Vries will join the Spain debrief by skype. These discussions will constitute the first formal ‘think-through’ of the whole week’s work, and as such will be a valuable moment for reflection. This session should be prepared for as far as possible, given time constraints, and we should be ready to discuss some of the main themes and issues that we may want to highlight in the respective country report.

#### Media and politics

Since we are representing ECDC as well as Umeå University, we should avoid talking to the media about our work – there is always the risk of misinterpretation. If the media approach us (and apparently, they might!), please refer them to the National Focus Point in the country where you are working – and also inform the PI in Umeå and Svetla Tsolova at ECDC. Similarly, we should avoid saying things during interviews or other interactions with the people we meet that may be seen as endorsing a particular political position. We should be seen only as friendly, neutral observers, and we must adhere to strict confidentiality about what we learn during data collection.

#### Receipts and reimbursement

In order to be reimbursed for any project-related costs (e.g. for transport etc.), we will need to save our receipts and submit them upon our return, as per standard practice for _____. Reimbursement rates for meals are fixed, so we don’t need to save these receipts. Flights will be booked through______. Boarding passes for flights should be saved and attached to our reimbursement applications.

#### After the trip

All the cleaned field notes should be completed and emailed securely to the team and to the project PI by the time we leave the country. These and the notes from the Friday debriefing sessions will constitute the basis for the country reports that each team will be writing. The structure of the reports remains to be worked out (in collaboration with ECDC and the country NFPs), but we will be looking to present best practices as well as any important gaps we have identified. We will agree on the report structures in advance and distribute templates in good time.

Draft reports should be produced in English, from each country team, within one month of the country visit. Subsequently, there will be several rounds of revision, based on input from ECDC and from the country NFPs.

Note that we will be able to add to and amend the material presented in the Country Reports once we put things together into the final Technical Report, but the Country Reports will nonetheless represent an essential ‘holding area’ for the key data and our initial analysis.

#### Questions arising during field work

If there are any uncertainties about anything during field work – scientific, logistic, or otherwise – it is important that we make contact with each other fast (i.e. either with the PI or, if the PI requires input, with Daniel de Vries) in order to obtain clarification. For issues that may require input from ECDC, immediately contact __________. Our field work time is short and we cannot afford to waste any of it.
